# The effect of positive psychology interventions on well-being and distress in clinical samples with psychiatric or somatic disorders: a systematic review and meta-analysis

**DOI:** 10.1186/s12888-018-1739-2

**Published:** 2018-06-27

**Authors:** Farid Chakhssi, Jannis T. Kraiss, Marion Sommers-Spijkerman, Ernst T. Bohlmeijer

**Affiliations:** 1 Scelta, GGNet, Apeldoorn, The Netherlands; 2 Centre for eHealth and Well-being Research, Department of Psychology, Health, and Technology, University of Twente, PO Box 217, 7500 AE Enschede, The Netherlands

**Keywords:** Well-being, Distress, Positive psychology, Chronic illness, Meta-analysis, Interventions

## Abstract

**Background:**

Although positive psychology interventions (PPIs) show beneficial effects on mental health in non-clinical populations, the current literature is inconclusive regarding its effectiveness in clinical settings. We aimed to examine the effects of PPIs on well-being (primary outcome), depression, anxiety, and stress (secondary outcomes) in clinical samples with psychiatric or somatic disorders.

**Methods:**

A systematic review and meta-analysis was conducted following PRISMA guidelines. PsycINFO, PubMed, and Scopus were searched for controlled studies of PPIs in clinical samples between Jan 1, 1998 and May 31, 2017. Methodological quality of each study was rated. We used Hedges’ adjusted g to calculate effect sizes and pooled results using random-effect models.

**Results:**

Thirty studies were included, representing 1864 patients with clinical disorders. At post-intervention, PPIs showed significant, small effect sizes for well-being (Hedges’ *g* = 0.24) and depression (*g* = 0.23) compared to control conditions when omitting outliers. Significant moderate improvements were observed for anxiety (*g* = 0.36). Effect sizes for stress were not significant. Follow-up effects (8–12 weeks), when available, yielded similar effect sizes. Quality of the studies was low to moderate.

**Conclusion:**

These findings indicate that PPIs, wherein the focus is on eliciting positive feelings, cognitions or behaviors, not only have the potential to improve well-being, but can also reduce distress in populations with clinical disorders. Given the growing interest for PPIs in clinical settings, more high quality research is warranted as to determine the effectiveness of PPIs in clinical samples.

**Trial registration:**

PROSPERO CRD42016037451

**Electronic supplementary material:**

The online version of this article (10.1186/s12888-018-1739-2) contains supplementary material, which is available to authorized users.

## Background

Positive psychology is a relatively new field that focuses on enhancing well-being and optimal functioning rather than ameliorating symptoms, and complements rather than replaces traditional psychology [1]. Common themes in positive psychology include savoring, gratitude, kindness, promoting positive relationships, and pursuing hope and meaning [2].

Now that it has been repeatedly shown that well-being and psychopathology are two moderately correlated yet independent constructs of mental health [3–6], well-being receives growing attention in clinical research and practice. Even after successful treatment of psychopathology, low levels of well-being may persist in individuals, which, in turn, form a substantial risk factor for psychological distress [7]. In the light of a substantial body of evidence demonstrating that high levels of well-being buffer against psychological symptomatology, including relapse or recurrence of symptoms, besides enhancing quality of life and longevity [5, 7–14], we anticipate that clinical samples could greatly benefit from positive psychological interventions (PPIs) which explicitly aim to enhance well-being, that is, positive feelings, cognitions or behaviors [15].

Although PPIs have been mostly examined in non-clinical samples [16], some preliminary evidence exist for their efficacy in clinical samples [16, 17]. Independent lines of research have shown that PPIs improved well-being and decreased psychological distress in mildly depressed individuals [18], in patients with mood and depressive disorders [19, 20], in patients with psychotic disorders [21] and improving quality of life and well-being in breast cancer patients [22]. Thus, PPIs may have the potential to be of value to clinical samples but their effectiveness in these samples is not well established.

To date, two meta-analyses have been published that examined the effectiveness of PPIs in predominantly non-clinical samples. First, Sin and Lyubomirsky [17] included 49 controlled studies with 4235 individuals examining the effectiveness of PPIs on well-being and depression. They found that PPIs were significantly more effective than comparators (i.e. active control or treatment as usual) for enhancing well-being (*r* = .29) and decreasing depression (*r* = .31). Second, to address several methodological issues in Sin and Lyubomirsky’s meta-analysis [17] such as lack of methodological quality assessment of the included studies, Bolier and colleagues [16] re-examined the literature. Using more stringent methodological and inclusion criteria, they systematically collected and synthesized the findings of 39 randomized controlled studies with 6139 individuals. Small but significant effects of PPIs on subjective well-being, psychological well-being and depression were found, with Cohen’s *d* effect sizes of 0.34, 0.20 and 0.23, respectively.

However, these previously published meta-analyses are inconclusive regarding the effectiveness of PPIs in improving well-being and alleviating psychological distress in clinical samples. Although both meta-analyses included a number of studies with clinical samples, 12 out of 49 studies [17] and 4 out of 39 studies [16], respectively, these were limited to psychiatric samples with depressive or anxiety symptoms. To our knowledge, no attempt has been made to systematically examine the effects of PPIs in samples with somatic disorders who may benefit from improvements in well-being [23].

Since there is growing interest in the application of PPIs targeting clinical samples, the aim of the study was to add to the existing literature on the effectiveness of PPIs in primarily non-clinical samples [16, 17] through meta-analytically testing the effects of PPIs on well-being and distress across a broad range of clinical samples with psychiatric and somatic disorders.

## Methods

This study was prepared and conducted according to the preferred reporting items for systematic reviews and meta-analyses (PRISMA) guidelines [24] and registered on April 29, 2016 in PROSPERO (#CRD42016037451), an international prospective register for systematic reviews.

### Search strategy

The electronic databases PsycINFO, PubMed, and Scopus were searched from 1998 (the start of the positive psychology movement) to March 31, 2016, and an update of the search was conducted on May 31, 2017. For each database, text word search terms, medical subject headings (PubMed) or thesaurus terms (PsycINFO) were used relating to ‘well-being’ and ‘positive psychology’, in combination with terms related to ‘interventions’, ‘disorders and illness’ and ‘outcome’ (see Additional file 1 for more detailed information on the search terms). Studies cited in the previously published meta-analytic reviews [16, 17, 22] were cross-checked. Additionally, three clinical trial registers (www.clinicaltrialsregister.eu, www.clinicaltrials.gov, www.isrctn.com) were searched on August 31, 2016, to detect trials with unpublished results available.

### Selection of studies

Potentially eligible studies were screened on title in the first phase, on abstract in the second phase, and on full paper in the third phase. Studies were included in the meta-analysis if they: 1) examined the effects of an intervention developed in line with the theoretical tradition of positive psychology cfm. Sin and Lyubomirsky (2009), that is, a psychological intervention (i.e. training, exercise, therapy) aimed at raising positive feelings, cognitions or behaviors; 2) included adult participants (18 years or older) with clinical psychiatric or somatic disorders [according to the International Classification of Diseases and Related Health Problems; [25]; 3) used an outcome measure of social, emotional or psychological well-being; 4) used a control condition; and 5) provided an effect size or sufficient information to calculate an effect size. Studies were excluded if they: 1) were not published in an English language peer-reviewed journal; 2) examined physical exercises aimed at improving well-being; or 3) used an intervention that is primarily based on reminiscence, mindfulness and/or meditation. With regard to the third exclusion criterion, extensive meta-analyses have already been published for these types of interventions [26–30]. Published abstracts and/or study protocols were also excluded.

The first (FC) and second author (JTK) independently conducted the screening of titles. The interrater reliability was high (kappa = 0.84; *n* = 1000). Disagreements between raters during the screening of abstracts and full texts were discussed until consensus was reached. Any remaining ambiguity was resolved with the third (MSS) and fourth author (ETB).

### Data extraction

Data were collected on: 1) population characteristics, including age, gender, disorder, and sample size (per condition); 2) intervention characteristics, including type of PPI, delivery mode, number of sessions, duration in weeks, retention rate, and guidance (i.e. with or without therapist); 3) methodological characteristics, including study design, type of control group, assessment points (i.e. pre, post and/or follow up), and outcome measures. Eight authors were contacted because information regarding study characteristics or to calculate effect sizes was lacking, of whom six provided additional data on request.

### Quality assessment

All studies were rated on methodological quality using criteria based on the Cochrane Collaboration’s tool for assessing risk of bias [31] and the Jadad scale [32]. This rating consists of seven items that are rated as 0 (“absent”) or 1 (“present”), resulting in a maximum quality score of 7 points. Studies were identified as “good” when all seven criteria were met, “fair” when five or six criteria were met, and “poor” when four or less criteria were met [33]. The included items cover: 1) random sequence generation and allocation concealment (i.e. sufficient description of the method used to generate and conceal the allocation sequence); 2) blinding of outcome assessments (i.e. outcome assessments are either administered online or by an independent person who is not involved in the study), 3) reporting incomplete outcome data (i.e. dropout analysis is conducted or reasons for drop-out are reported), 4) using intention-to-treat analysis, 5) group similarity at baseline regarding prognostic factors (e.g. demographics) or adjustments were made to correct for baseline imbalance, 6) adequate sample size/power analysis (i.e. an adequate power analysis was conducted or the study included 50 or more persons in the analysis), and 7) reliability of the diagnostic assessment (i.e. assessment was conducted by a professional and not based on self-report or screening or there were no diagnostic assessments). The first (FC) and second author (JTK) independently conducted the quality assessment, whereby disagreements were discussed until consensus was reached.

### Primary and secondary outcomes

The primary outcome was the mean well-being score at the end of the intervention, assessed with validated measures of social, emotional, and/or psychological well-being. In the absence of well-being measures, measures, constructs related to well-being such as hope, happiness, life satisfaction, personal growth, optimism or positive affect were included if available. If more than one measure for well-being was used, we used the most validated measure, to ensure each study had one primary outcome for the analysis. Secondary outcomes included depression, anxiety and stress.

### Statistical analysis

For each study, means and standard deviations were extracted, where possible based on the intention-to-treat method; otherwise, the reported means and standard deviations for the patients that completed the interventions were used. Effect sizes were calculated in three steps. First, standardized pre-post effect sizes were calculated per condition (i.e. PPI or control condition) by subtracting the average pre-intervention score from the average post-intervention score and subsequently dividing this score by the pooled standard deviation. Second, the difference in effect size (Δ*d*) between PPI condition and control condition was computed. Third, Δ*d* was adjusted for small sample bias, indicated as Hedges’ *g*. Where possible, pre-to-follow-up effect sizes were calculated in a similar manner, thereby only using studies with a follow-up period between 8 and 12 weeks.

Using Comprehensive Meta-Analysis version 2.2.064, separate meta-analyses were performed for 1) well-being, 2) depression, 3) anxiety, and 4) stress in which data were pooled using the random-effects model accounting for diversity across studies (e.g. in terms of populations, types of PPIs and outcome measures). Effect sizes of 0.56 to 1.2 can be considered large, effect sizes of 0.33 to 0.55 moderate, and effect sizes of 0 to 0.32 small [34].

Heterogeneity of effect sizes was examined using *Q* and *I*^*2*^ statistics. The Q-test assesses whether the observed effect sizes are significantly more different from one another than would be expected based on chance alone. A significant Q-statistic indicates heterogeneity. The *I*^*2*^ statistic captures the percentage of the total variance across the included studies attributable to heterogeneity. A value of zero indicates true homogeneity, while values of 25, 50, and 75% indicate low, moderate, and high levels of heterogeneity, respectively [35].

Publication bias was assessed using funnel plots, Egger’s Test, Duval and Tweedie’s trim-and-fill procedure, and fail-safe N. First, a funnel plot was created by plotting the overall mean effect size against study size. Whereas a symmetric distribution of studies around the effect size indicates the absence of publication bias, a higher concentration of studies on one side of the effect size than on the other indicates publication bias [36]. Second, Egger’s test [37] was used to examine the symmetric distribution of studies around the effect size with a quantitative test statistic (considered significant funnel plot asymmetry if *p* < 0.05). Third, Duval and Tweedie’s [38] trim-and-fill procedure was applied. This procedure imputes the effect sizes of missing studies and produces an adjusted effect size accounting for these missing studies. Adjusted values were only reported for pooled effect sizes when these were statistically significant. Finally, a fail-safe N, a test of funnel plot asymmetry, was calculated for each analysis. The fail-safe N indicates the number of unpublished non-significant studies that would be required to lower the overall effect size below significance [37]. The findings were considered robust if the fail-safe *N* ≥ 5n + 10, where n is the number of comparisons [39].

Pre-specified exploratory subgroup analyses were performed to examine differences in effect sizes based on: 1) population type: psychiatric vs somatic disorders; 2) intervention type: individual vs. group format, with vs. without therapist guidance; and 3) duration of the intervention: short (≤ 8 weeks) vs long (> 8 weeks). Mixed effects analysis was used to tests for differences between subgroups. Additional ad hoc analyses were performed to explore differences in effect sizes based on: 1) type of PPI: PPI therapy programs (e.g. meaning-centered group approach, well-being therapy) vs single PPIs (e.g. three good things/signature strengths); and 2) control group: no intervention (i.e. did not receive any intervention at all)/waitlist (i.e. did receive the intervention after the experimental group) vs. active/treatment-as-usual. Finally, meta-regression analysis was performed to investigate if effect sizes were moderated by study quality.

## Results

### Selection of studies

**Fig. 1:**
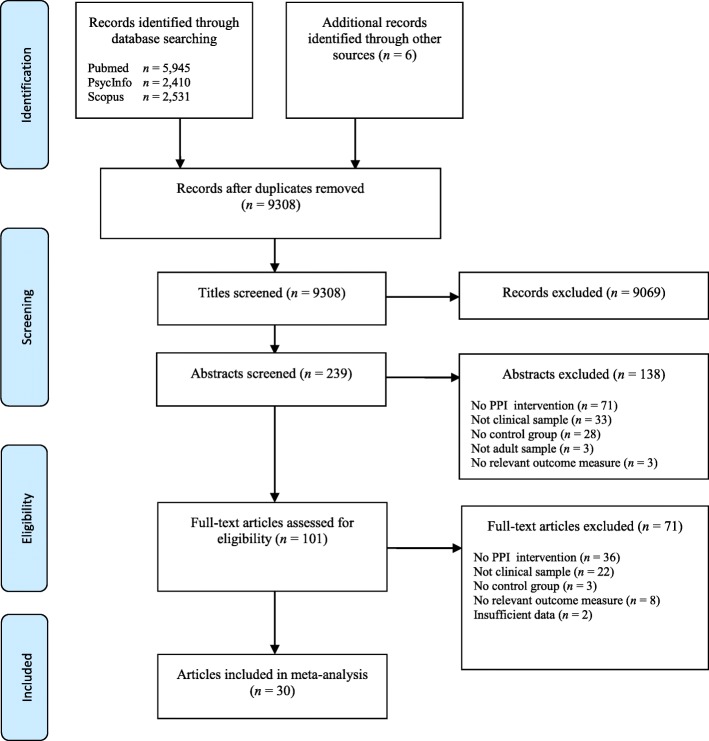
Flowchart of the study selection process

A total of 10,886 studies were produced in the electronic database searches. After the exclusion of duplicates (*n* = 1578) and the removal of studies at the title screening phase (*n* = 9069), 239 abstracts were reviewed (Fig. 1). Of the 101 articles identified for full text review, 30 controlled studies were included. The 30 studies comprised 33 comparisons for wellbeing, 26 comparisons for depression, 14 comparisons for anxiety and 6 comparisons for stress [40–69]. Fourteen studies were conducted in the United States, three each in Iran, Canada, and Spain, two each in the United Kingdom and Italy, and one each in Australia, Germany, and Taiwan. The characteristics of the included studies are presented in Table 1.

**Table 1:** Characteristics of studies included in the systematic review and meta-analysis

First author (Year)	Disorder	% female	Mean age (SD)	PPI name (*n*)	Format (guidance)	Duration in days or weeks (n sessions)	Control group (*n*)	Retention rate post-treatment		Follow-up (in weeks)	Outcome measure			
								PPI	Control		WB	DEP	ANX	S
Andrewes (2014) [40]	Brain injury	10%	42.2 (8.5)	Three good things in life / Signature strengths (4)	Individual (Yes)	2w (1–2)	CBT (5)	80%	100%	10	AHI			
Asgharipoor (2012) [41]	Major Depressive Disorder	72%	26.4 (5.9)	Positive psychotherapy (9)	Group (Yes)	12w (12)	CBT (5)	100%	100%	–	EWBS	BDI-II		SUDS
Breitbart (2010) [43]	Advanced cancer	51%	60.1 (11.8)	Meaning-centered group psychotherapy (49)	Group (Yes)	8w (8)	Supportive Psychotherapy (41)	71.4%	48.8%	8	FACIT-SP	HADS-D	HADS-A	
Breitbart (2012) [42]	Advanced cancer	61%	54.4 (11.6)	Meaning-centered group psychotherapy (64)	Individual (Yes)	7w (7)	Massage (56)	64.1%	66.1%	8	FACIT-SP	HADS-D	HADS-A	
Breitbart (2015) [44]	Advanced cancer	70%	58.2 (11.0)	Meaning-centered group psychotherapy (132)	Group (Yes)	8w (8)	Supportive Psychotherapy (121)	52.3%	47.9%	8	FACIT-SP	BDI-II	HADS-A	
Celano (2016) [55]	Major Depressive Disorder	69%	44 (16.6)	Positive Psychology Intervention (32)	Individual (Yes)	6w (6)	Cognition Focused Intervention (33)	90.6%	87.8%	6	PA	QIDS-SR		
Cerezo (2014) [46]	Breast cancer	100%	50 (9.7)	Positive Psychology Intervention (87)	Group (Yes)	14w (14)	Waitlist (88)	86.1%	83%	–	SWLS			
Chaves (2017) [47]	Depression/ Dysthymia	100%	51.7 (10.4)	Positive Psychology Intervention (47)	Group (Yes)	10w (10)	CBT (49)	72.4%	79.6%	–	SWLS	BDI-II	BAI	
Cohn (2014) [48]	Type 2 diabetes	51%	54 (U)	DAHLIA: Developing Affective HeaLth to Improve Adherence (29)	Individual (No)	5w (0)	Emotion reporting (20)	86.2%	85%	–	PA	CES-D		PSS
Coote (2012) [49]	Depression	71%	52.5 (13.4)	Goal-setting and Planning (26)	Individual (No)	5w (0)	Waitlist (29)	92.3%	62.1%	5	PA	CES-D		
Elham (2015) [50]	Cardiovascular diseases	41%	68.9 (8.3)	Need-based spiritual/religious interventions (33)	Individual (Yes)	3 days (3)	No treatment (33)	100%	100%	–	SWBS		STAI	
Fava (1998) [51]	Affective disorders	55%	28.4 (6.6)	Well-being therapy (10)	Group (Yes)	16w (8)	CBT (10)	100%	100%	–	PWB	CID-D	SQ-A	
Fava (2005) [52]	Generalized anxiety disorder	65%	41.9 (11.9)	Well-being therapy (10)	Group (Yes)	16w (8)	CBT (10)	80%	80%	52	PWB	CID-D	CID-A	
Henry (2010) [53]	Stage III or IV ovarian cancer	100%	55 (9.7)	The Meaning-Making intervention (12)	Individual (Yes)	8w (3)	TAU (12)	80%	92.3%	12	FACIT-SP	HADS-D	HADS-A	
Hsiao (2012) [54]	Breast cancer	100%	46.2 (8.6)	Body-mind-spirit (BMS) group therapy (26)	Group (Yes)	8w (8)	One psycho-educational session (22)	69.2%	95.4%	32	MLQ	BDI-II		
Huffman (2016) [55]	Coronary syndrome	40%	62.8 (11.5)	Positive Psychology Interventions (23)	Individual (Yes)	8w (8)	TAU (25)	87%	88%	–	PA	HADS-D	HADS-A	
Kent (2011) [56]	Posttraumatic Stress Disorder	33%	54 (8.34)	Resilience-Oriented Treatment (20)	Group (Yes)	12w (12)	Waitlist (19)	95%	89.5%	–	PWB	BDI-II	STAI	PDS
Kerr (2015) (Group 1 gratitude)	Various mental problems	75%	43 (11.1)	Gratitude Interventions (16)	Individual (No)	2w (14)	Mood monitoring (15)	?	?	–	MLQ	DASS-D	DASS-A	DASS-S
Kerr (2015) (Group 2 Kindness)	Various mental problems	75%	43 (11.1)	Kindness Interventions (16)	Individual (No)	2w (14)	Mood monitoring (15)	?	?	–	MLQ	DASS-D	DASS-A	DASS-S
Krentzman (2015) [58]	Alcohol use disorder	48%	46.3 (10.9)	Web-based gratitude exercise (11)	Individual (No)	2w (14)	Placebo (11)	?	?	8	PA			
Lee (2006) [59]	Breast or colorectal cancer	81%	56.7 (10)	Meaning-making intervention (35)	Individual (Yes)	4.5w (4)	No treatment (39)	85.4%	95.1%	–	LOT-R			
Louro (2016) [60]	Colorectal cancer	34%	(U)	Enhancing Positive Emotions Procedure (31)	Group (Yes)	6w (4)	No treatment (21)	77.4%	95.2%	4	PA			
Mann (2001) [61]	HIV patients	100%	38.5 (8.2)	Future Writing and Optimism (21)	Individual (No)	4w (8)	No treatment (23)	95.2%	87%	–	LOT-R			
Muller (2016) [62]	Physical disability and chronic pain	70%	59.4 (11.8)	Computer-based positive psychology intervention (51)	Individual (No)	8w (0)	Writing exercises (45)	76.5%	74.5%	10	PWI	HADS-D		
Nikrahan (2016) [63] (group 1)	Heart diseases	24%	56.6 (8.7)	Fordyce’s positive CBT (15)	Group (Yes)	6w (6)	Waitlist (14)	66.7%	85.7%	–	SWLS	BDI-II		
Nikrahan (2016) [63] (group 2)	Heart diseases	24%	56.6 (8.7)	Lyubomirsky’s the how of happiness (13)	Group (Yes)	6w (6)	Waitlist (14)	92.3%	85.7%	–	SWLS	BDI-II		
Nikrahan (2016) [63] (group 3)	Heart diseases	24%	56.6 (8.7)	Seligman’s authentic Happiness (13)	Group (Yes)	6w (6)	Waitlist (14)	76.9%	85.7%	–	SWLS	BDI-II		
Pietrowsky (2012) [64]	Depression	53%	38.9 (8.6)	Positive Psychology Interventions (9)	Group (Yes)	4w (3)	TAU (8)	77.8%	75%	–	SWLS	BDI-II		
Sanjuan (2016) [65]	Cardiac diseases	18%	54.4 (9.1)	Program to improve Well-being (57)	Group (Yes)	6w (24)	Relaxation (51)	87.7%	84.3%	–	PA	SCL90-D		
Schrank (2016) [66]	Psychosis	40%	42.5 (11.3)	Positive psychotherapy (47)	Group (Yes)	11w (11)	TAU (47)	91.5%	87.2%	–	WEMWBS	SDHS		
Seligman (2006) [67] (study 2)	Depression	76%	(U)	Positive psychotherapy (11)	Individual (Yes)	12w (14)	TAU (9)	84.6%	60%	–	SWLS	ZSRS		
Taylor (2017) [68]	Depression / Anxiety	62.5%	29.4 (12.1)	Positive Activity Intervention (16)	Individual (Yes)	10w (10)	Waitlist (12)	100%	92.3%	–	SWLS	BDI-II	STAI	
Uliaszek (2016) [69]	Severe emotion dysregulation	78%	22.2 (5.0)	Positive Psychotherapy	Group (Yes)	12w (12)	DBT	55.6%	85.2%	–	PPTI	SCL-90-D	SCL-R-A	DTS

*Note. AHI* Authentic Happiness Index, *ANX* Anxiety, *BAI* Beck Anxiety Inventory, *BDI-II* Beck Depression Inventory-II, *CES-D* Center for Epidemiologic Studies Depression Scale, *CID-A* Clinical Interview for Depression - Anxiety subscale, *CID-D* Clinical Interview for Depression - Depression subscale, *EWBS* Emotional Well-Being Scale, *DBT* Dialectical Behaviour Therapy, *DEP* Depression, *DTS* Distress Tolerance Scale, *FACIT-SP* Functional Assessment of Chronic Illness Therapy - Spiritual Well-being Scale, *HADS* Hospital Anxiety and Depression Scale, *HADS-A* Hospital Anxiety and Depression Scale – Anxiety Scale, *HADS-D* Hospital Anxiety and Depression Scale - Depression Scale, *HS* The Hope Scale, *LOT-R* Life Orientation Test – Revised, *MLQ* The Meaning in Life Questionnaire, *PA* The Positive and Negative Affect Scale - Positive Affect Scale, *PDS* Posttraumatic Stress Diagnostic Scale, *PPI* Positive Psychological Intervention, *PPTI* Positive Psychotherapy Inventory, *PSS* Perceived Stress Scale, *PWB* The Ryff Scales of Psychological Well-Being, *PWBS* Psychological Well-Being Scale, *PWI* Personal Well-being Index, *QIDS-SR* Quick Inventory of Depressive Symptomatology, Self-Report, *S* Stress, *SCL-90-A* Symptom Checklist 90 – Anxiety subscale, *SCL-90-D* Symptom Checklist 90 - Depression subscale, *STAI* Spielberger State – Trait Anxiety Inventory, *SQ-A* Symptom Questionnaire - Anxiety subscale, *SUDS* Subjective Units of Distress Scale, *SWB* Subjective Well-Being, *SWBS* Spiritual Well-Being Scale, *SWLS* Satisfaction with Life Scale, *TAU* Treatment-as-Usual, *U* Unknown, *WB* Well-being, *WEMMWB* Warwick-Edinburgh Mental Well-Being Scale

### Population characteristics

The included studies comprised 1864 adult participants, 960 in the PPI conditions and 904 in the control conditions. The mean age of the participants at pre-intervention was 47.8 years (*SD* = 11.5, range 26.4–68.9), and more than half were women (61.5%). In 16 studies, clinical samples with somatic disorders were included, with cancer being the most prevalent disorder (8 out of 16 studies). Other somatic disorders included cardiac diseases (*n* = 4), HIV (*n* = 1), brain injuries (*n* = 1), diabetes (*n* = 1) and chronic pain (*n* = 1). The remaining 14 studies included samples with psychiatric disorders, with depressive disorder as the most prevalent disorder (7 out of 13 studies), followed by anxiety disorders (*n* = 2), severe emotion dysregulation (*n* = 1), psychotic disorders (*n* = 1), post-traumatic stress syndrome (*n* = 1), and various mental health problems (*n* = 2).

### Intervention, comparison and outcome characteristics

In 20 studies, PPIs were compared to treatment as usual or an active control condition, such as supportive psychotherapy [44], cognitive behavioral therapy [47], dialectical behavior therapy [69] or mood monitoring [57]. Ten studies compared PPIs to a no intervention/waitlist condition. The names of the PPIs as provided by the authors of the studies are also displayed in Table 1. All interventions were explicitly aimed at raising positive feelings, cognitions or behaviors. The 24 studies used empirically validated PPIs (see [2, 18]) or programs that have incorporated PPIs such as positive psychotherapy [67] or wellbeing therapy [51]. In 24 studies, therapist guidance was part of the PPI. The intervention duration varied from 3 days to 16 weeks. The mean retention rate, based on dropouts at post-intervention, was 81.4% (available for 26 studies). For the PPI conditions, the mean retention rate was 81.0% and for the control conditions 81.8%. For the 12 studies that included follow-up measurements, the average follow-up time was 12.9 weeks after post-intervention.

### Quality of studies

The quality scores of the studies are displayed in Table 2. If a criterion was not reported in the paper, it was labeled “unclear”, and the criterion was rated as not met. All studies were either of medium quality (*n* = 12) or of low quality (*n* = 18). None of the included studies met all quality criteria. The use of intention-to-treat analyses was the most poorly rated, with only 11 studies meeting this criterion.

**Table 2:** Methodological quality of studies included in the meta-analysis

First author (year)	1. Adequate allocation sequence generation and allocation concealment	2. Blinding of main outcome assessments	3. Description of withdrawals/drop-outs	4. Intention-to-treat analysis is performed or there are no drop-outs	5. The sample size is based on an adequate power analysis.	6. The groups are similar on prognostic indicators at baseline (and this was explicitly assessed) or adjustments were made to correct for baseline imbalance (using appropriate covariates).	7. Diagnostic assessment was conducted by a professional, or there were no diagnostic assessments necessary for the recruitment	Score
Andrewes (2014) [40]	Yes	No	Yes	No	No	Yes	Yes	4
Asgharipoor (2012) [41]	Unclear	No	Unclear	Unclear	No	Yes	Yes	2
Breitbart (2010) [43]	Unclear	Unclear	Yes	No	Yes	Yes	Yes	4
Breitbart (2012) [42]	Yes	Unclear	Yes	No	Yes	Yes	Yes	5
Breitbart (2015) [44]	Unclear	Unclear	Yes	No	Yes	Yes	Yes	4
Celano (2016) [55]	Yes	Yes	No	Yes	Yes	Yes	Yes	6
Cerezo (2014) [46]	Yes	Unclear	No	No	Yes	Yes	Yes	4
Chaves (2017) [47]	Unclear	Yes	Yes	Yes	Yes	Yes	Yes	6
Cohn (2014) [48]	Yes	Yes	No	No	Yes	Yes	Unclear	4
Coote (2012) [49]	Unclear	Yes	No	No	Yes	Yes	No	3
Elham (2015) [50]	No	No	Yes	Yes	Yes	Yes	Yes	5
Fava (1998) [51]	Unclear	Yes	Yes	Yes	No	Unclear	Yes	4
Fava (2005) [52]	Unclear	Yes	No	No	No	Unclear	Yes	2
Henry (2010) [53]	Yes	Yes	Yes	No	No	Yes	Yes	5
Hsiao (2012) [54]	Yes	Unclear	Yes	No	No	Yes	Yes	4
Huffman (2016) [55]	No	No	Yes	No	No	Yes	Yes	3
Kent (2011) [56]	Unclear	Unclear	Yes	Yes	No	Yes	Yes	4
Kerr (2015)	Unclear	Unclear	Yes	Yes	No	Yes	Yes	4
Krentzman(2015) [58]	Unclear	Yes	No	No	No	Yes	Yes	3
Lee (2006) [59]	Yes	Yes	Yes	No	Yes	Yes	Yes	6
Louro (2016) [60]	No	No	Yes	No	No	Yes	Yes	3
Mann (2001) [61]	Unclear	No	No	No	No	Yes	Yes	2
Muller (2016) [62]	Yes	Yes	Yes	No	Yes	Yes	Yes	6
Nikrahan (2016) [63]	Unclear	Unclear	Yes	Yes	Yes	Yes	Yes	5
Pietrowsky (2012) [64]	Unclear	No	No	Yes	No	Yes	Yes	3
Sanjuan 2016 [65]	Yes	Unclear	Yes	No	Yes	Yes	Yes	5
Schrank (2016) [66]	Yes	No	Yes	Yes	Yes	Yes	Yes	6
Seligman (2006) [67] (study 2)	Unclear	Yes	Yes	No	No	Yes	Yes	4
Taylor (2017) [68]	Yes	Unclear	Yes	Yes	No	Yes	Yes	5
Uliaszek (2016) [69]	Yes	No	No	Yes	Yes	Yes	Yes	5

### Meta-analyses

**Fig. 2:**
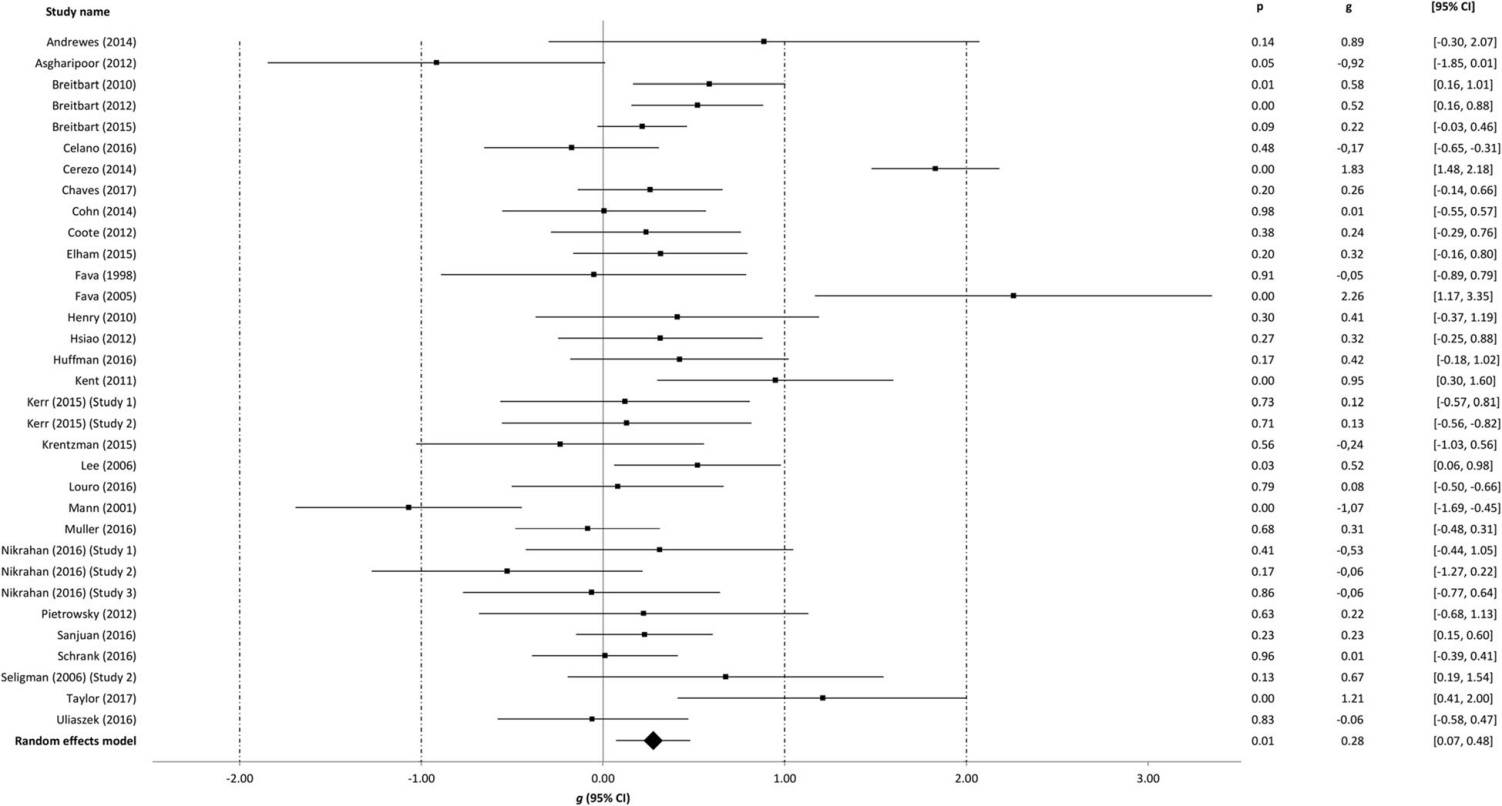
Post-intervention effects of positive psychology interventions on well-being. The square boxes show Hedges’ g effect size and sample size (the larger the box, the larger the sample size) in each study, and the line the 95% confidence interval. The diamond reflects the pooled effect size and the line the width of the 95% confidence interval

**Fig. 3:**
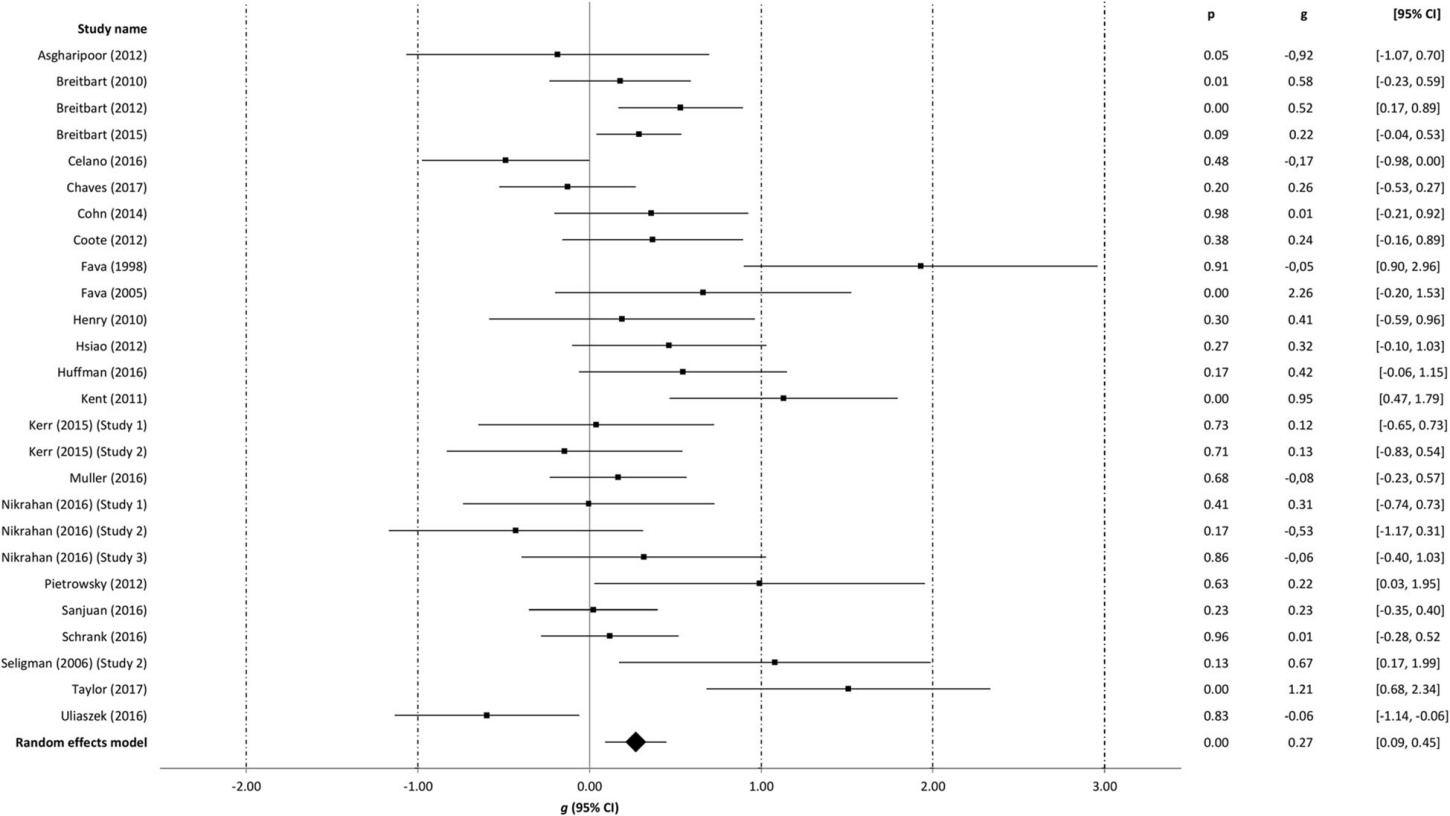
Post-treatment effects of positive psychology interventions on depression. The square boxes show Hedges’ g effect size and sample size (the larger the box, the larger the sample size) in each study, and the line the 95% confidence interval. The diamond reflects the pooled effect size and the line the width of the 95% confidence interval

**Fig. 4:**
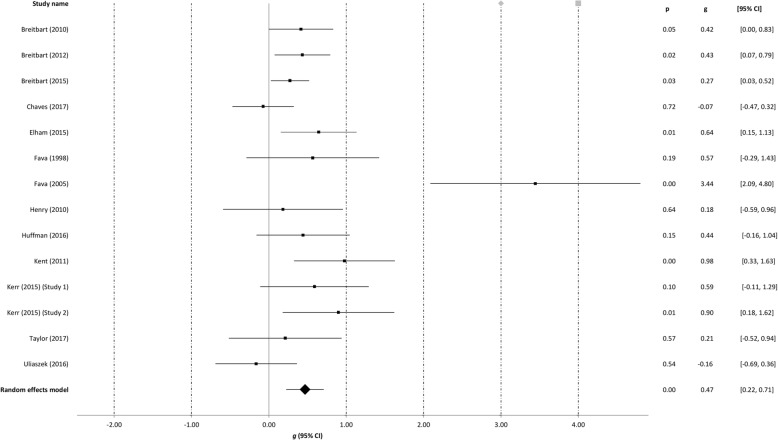
Post-treatment effects of positive psychology interventions on anxiety. The square boxes show Hedges’ g effect size and sample size (the larger the box, the larger the sample size) in each study, and the line the 95% confidence interval. The diamond reflects the pooled effect size and the width of the 95% confidence interval

**Fig. 5:**
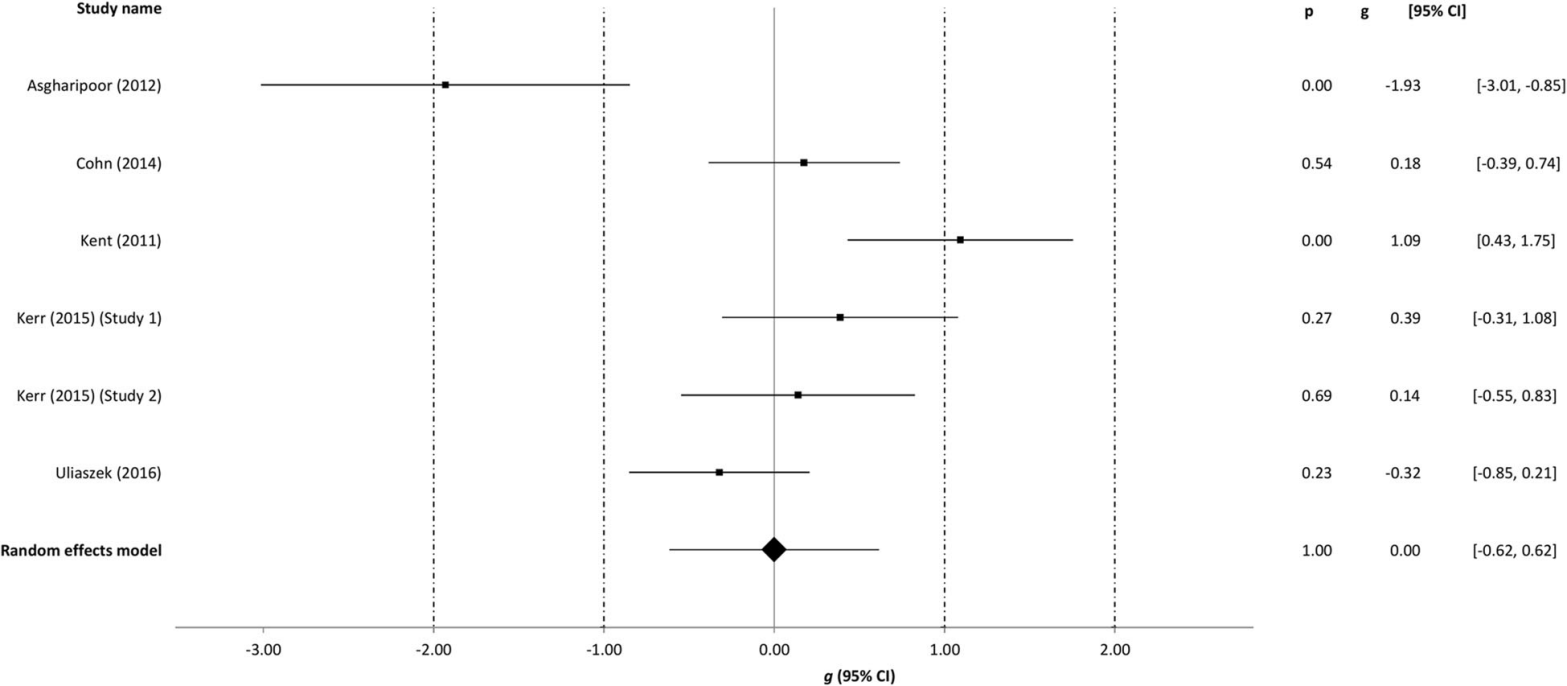
Post-treatment effects of positive psychology interventions on stress. The square boxes show Hedges’ g effect size and sample size (the larger the box, the larger the sample size) in each study, and the line the 95% confidence interval. The diamond reflects the pooled effect size and the width of the 95% confidence interval

Table 3 summarizes findings from the meta-analyses per outcome, i.e. well-being, depression, anxiety, and stress. The meta-analyses were run separately for all studies at post-intervention with the outliers included, with the outliers excluded and with the low quality omitted. The meta-analyses at follow-up were run including outliers and low quality studies. The effect sizes of the individual studies at post-intervention are plotted in Figs. 2, 3, 4 and 5.

**Table 3:** Between-group effects

Outcome measures	*N*_comp_	Hedges’ *g*	95% CI	*Z*	Heterogeneity		Fail-safe *N*
					*Q*-value	*I* ^*2*^	
All studies post-treatment (including outliers)							
Well-being	33	0.28	0.07–0.48	2.66**	146.81***	78.20	271
Depression	26	0.27	0.09–0.45	2.97**	66.40***	62.34	132
Anxiety	14	0.47	0.23–0.71	3.78***	36.83***	64.71	135
Stress	6	0.00	-0.62–0.62	0.00	25.35***	80.28	0
All studies post-treatment (excluding outliers)^a^							
Well-being	29	0.24	0.13–0.35	4.16***	35.13	20.29	137
Depression	21	0.23	0.11–0.34	3.74***	22.26	10.16	66
Anxiety	13	0.36	0.20–0.53	4.24***	16.96	29.26	81
Stress	5	0.27	−0.19–0.73	1.16	11.02	63.69	0
Medium or high quality studies post-treatment							
Well-being	14	0.19	0.02–0.37	2.17*	21.99	40.88	17
Depression	12	0.07	-0.19–0.32	0.53	32.43	66.08	0
Anxiety	6	0.22	−0.05–0.49	1.57	8.39	40.39	1
Stress	1	−0.32	− 0.85–0.21	−1.19	0.00	0.00	–
Studies with 8–12 week follow up (including outliers)							
Well-being	7	0.41	0.08–0.74	2.46*	19.24**	68.82	28
Depression	5	0.21	0.05–0.37	2.53*	2.55	0.00	4
Anxiety	4	0.35	0.12–0.59	2.91**	4.45	32.54	10
Stress	–	–	–	–	–	–	–

*Note. N*_comp_, number of comparisons, *CI* confidence interval. *p < 0.05. ** *p* < 0.01. *** *p* < 0.001. ^***a***^ The effect size for wellbeing (*g* = 0.24) corresponds with a standardized mean difference Cohen’s *d* = 0.24 and unweighted mean *r* = 0.12; the effect size for depression (*g* = 0.23) corresponds with *d* = 0.23 and *r* = 0.11; the effect size for anxiety (g = 0.36) corresponds with *d* = 0.37 and *r* = 0.18; the effect size for stress (*g* = 0.27) corresponds with *d* = 0.28 and *r* = 0.14

### Post-intervention effects on well-being

For well-being (33 comparisons), a significant, small effect was observed (*g* = 0.28, 95% CI: 0.07 to 0.48, *p* = 0.008) at post-intervention. The level of heterogeneity was high (*I*^*2*^ = 78.20). Four outliers were detected [41, 46, 52, 61]. After omitting these studies from the analysis, we found a similar effect, with *g* = 0.24 (95% CI: 0.13 to 0.35, *p* < 0.001), and heterogeneity reduced substantially (*I*^*2*^ = 20.29). When studies scored as low quality were excluded from the analysis (including outliers), again a small significant effect size was observed (*g* = 0.19, 95% CI: 0.02 to 0.37, *p* = 0.030), with a moderate level of heterogeneity (*I*^*2*^ = 40.88).

### Post-intervention effects on depression

Based on 26 comparisons, we found a significant, small effect of PPIs on depression, with *g* = 0.27 (95% CI: 0.09 to 0.45, *p* = 0.003) at post-intervention. The level of heterogeneity was moderate (*I*^*2*^ = 62.34). Five outliers were detected [45, 51, 56, 68, 69]. After removal of the outliers, a small effect size was observed (*g* = 0.23, 95% CI: 0.11 to 0.34, *p* < 0.001). The level of heterogeneity was low (*I*^*2*^ = 10.16). After removal of low quality studies, the effect size for depression was not significant with *g* = 0.07 (95% CI: -0.19 to 0.32, *p* = 0.598), and heterogeneity was moderate (*I*^*2*^ = 66.08).

### Post-intervention effects on anxiety

For anxiety (14 comparisons), a significant, moderate effect was found (*g* = 0.47, 95% CI: 0.23 to 0.71, *p* < 0.001) at post-intervention. Heterogeneity was moderate (*I*^*2*^ = 62.34), and one outlier was detected [52]. After removal of the outlier, the effect size dropped to *g* = 0.36 (95% CI: 0.20 to 0.53, *p* < 0.001), but still remained in the moderate range, and the level of heterogeneity was low (*I*^*2*^ = 10.16). After removal of low quality studies from the analysis, the effect size for anxiety was small and not significant (*g* = 0.22, 95% CI: -0.05 to 0.49, *p* = 0.233), with moderate heterogeneity (*I*^*2*^ = 40.39).

### Post-intervention effects on stress

The overall mean effect size for 5 comparisons on stress was not significant (*g* = 0.00; 95% CI: -0.62 to 0.62, *p* = 0.999) at post-intervention. After the removal of one outlier [41], the effect size increased to the small range (*g* = 0.27; 95% CI: -0.19 to 0.73, *I*^*2*^ = 43.89) but remained non-significant (*p* = .247). Only 1 study that included stress as an outcome had a medium quality rating (see Table 1).

### Effects at follow-up

At follow-up, a significant, moderate effect was observed for well-being (*g* = 0.41, 95% CI: 0.08 to 0.74, *p* = 0.014), a significant, small effect for depression (*g* = 0.21, 95% CI: 0.05 to 0.37, *p* = 0.011), and a significant, moderate effect for anxiety (*g* = 0.35, 95% CI: 0.12 to 0.59, *p* = 0.004). There were no follow-up assessments conducted between 8 to 12 weeks with stress as outcome.

### Subgroup analyses

Exploratory subgroup analyses are presented in Table 4. For well-being (*Q* = 6.412, *df* = 1, *p* = 0.011) a significantly higher effect size was found for PPIs with therapist guidance (*g* = 0.39) than for PPIs without therapist guidance (*g* = − 0.12). For stress, PPIs were found significantly more effective in studies using a no intervention/waitlist control condition (*g* = 1.12 vs *g* = − 0.21; *Q* = 8.283, *df* = 1, *p* = 0.004) than in studies using an active or treatment-as-usual control condition. Effect sizes did not significantly vary based on population type (i.e. psychiatric vs somatic disorders), intervention format (i.e. individual vs group), intervention duration (i.e. shorter vs longer than 8 weeks) and/or type of PPI (i.e. PPI therapy programs vs single PPIs). For depression and anxiety, no significant differences between subgroups were found.

**Table 4:** Subgroup analyses (including outliers)

Outcome measure	Criterion	Subgroup	*N*_comp_	Hedges’ *g*	95% CI	*I* ^*2*^	*Z*
Well-being	Duration	<= 8 weeks	22	0.15	0.00–0.30	42.23	1.95
		> 8 weeks	11	0.59	0.06–1.11	88.74	2.19*
	Format	Group	17	0.33	0.01–0.66	84.87	2.01*
		Individual	16	0.20	−0.03–0.43	58.89	1.74
	Guidance	Therapist	26	0.39	0.16–0.62	78.63	3.30***
		Without therapist	7	−0.12	−0.43–0.20	49.96	−0.73
	Disorder	Psychiatric	15	0.26	−0.02–0.53	62.56	1.83
		Somatic	18	0.28	−0.01–0.57	83.99	1.91
Depression	Duration	<= 8 weeks	16	0.18	0.03–0.33	27.56	2.38*
		> 8 weeks	10	0.54	0.08–0.99	80.02	2.31*
	Format	Group	15	0.23	−0.01–0.47	64.37	1.91
		Individual	11	0.33	0.04–0.61	61.60	2.26*
	Guidance	Therapist	21	0.31	0.09–0.52	68.95	2.74**
		Without therapist	5	0.19	−0.05–0.43	0.00	1.55
	Disorder	Psychiatric	14	0.37	0.01–0.73	76.94	2.03*
		Somatic	12	0.26	0.13–0.39	0.00	3.86***
Anxiety	Duration	<= 8 weeks	7	0.41	0.25–0.57	0.00	5.02***
		> 8 weeks	7	0.59	0.03–1.16	81.11	2.05*
	Format	Group	7	0.52	0.08–0.96	81.15	2.31**
		Individual	7	0.49	0.28–0.70	0.00	4.53***
	Guidance	Therapist	12	0.43	0.17–0.70	67.87	3.21**
		Without therapist	2	0.74	0.24–1.24	0.00	2.89**
	Disorder	Psychiatric	8	0.65	0.12–1.18	79.75	2.41**
		Somatic	6	0.38	0.21–0.54	0.00	4.56***
Stress	Duration	<= 8 weeks	3	0.23	−0.14–0.59	0.00	1.20
		> 8 weeks	3	−0.33	−1.77–1.11	91.69	−0.45
	Format	Group	3	−0.33	−1.77–1.11	91.69	−0.45
		Individual	3	0.23	−0.14–0.59	0.00	1.20
	Guidance	Therapist	3	−0.33	−1.77–1.11	91.69	−0.45
		Without therapist	3	0.23	−0.14–0.59	0.00	1.20
	Disorder	Psychiatric	5	−0.06	−0.84–0.73	84.16	−0.14
		Somatic	1	0.18	−0.39–0.74	0.00	0.61

*Note. N*_comp_, number of comparisons; *CI* confidence interval. *p < 0.05. ** *p* < 0.01. *** *p* < 0.001

### Meta-regression analysis

Using meta-regression analysis, we found no evidence that effect sizes for well-being and stress were moderated by study quality. The study quality had a significant negative influence on the effect size for depression and anxiety, with lower study quality scores resulting in lower effect sizes for depression (slope: -0.17, *Z* = − 3.23, *p* = 0.001) and anxiety (slope: -0.28, *Z* = − 3.25, *p* = 0.001).

### Publication bias

First, inspection of the funnel plots showed that only for stress the funnel plot was skewed in favor of studies with a positive outcome at post-intervention. Second, Egger’s test statistic showed no significant funnel plot asymmetry for all analyses (all *p*-values > .05). Third, after adjusting for potential publication bias with Duval and Tweedie’s trim-and-fill procedure, the effect sizes for well-being and stress remained the same. However, for depression, four studies were trimmed and the adjusted effect size was *g* = 0.15 (95% CI: 0.05 to 0.25). Also for anxiety, four studies were trimmed and the adjusted effect size was *g* = 0.27 (95% CI: 0.14 to 0.39). Finally, the fail-safe N indicated that the findings for well-being and anxiety were robust, whereas the fail-safe numbers for depression (132) and stress (0) were lower than required (140 and 35, respectively). After omitting outliers, the findings for anxiety remained robust. The fail-safe N for well-being (137), depression (66) and stress (0) were lower than required (respectively 155, 115 and 35). At follow-up, the fail-safe N for well-being (28), depression (4) and anxiety (10) were lower than required (respectively 45, 35, and 30).

## Discussion

To our knowledge, this is the first meta-analysis examining the effects of PPIs on well-being and distress in clinical samples with psychiatric and somatic disorders. When excluding outliers, our analyses suggest that PPIs have a small but significant effect on well-being compared to control conditions. At follow-up, a significant moderate effect size of PPIs on well-being was observed. For the secondary outcomes, a small but significant effect size was found for depression at post-intervention and follow-up and moderate significant effect sizes for anxiety at post-intervention and follow-up. Effect sizes for stress were not significant. These findings suggest that PPIs not only have the potential to improve well-being, but can also reduce distress in populations with clinical disorders.

The effect sizes at post-intervention and follow-up for well-being and distress were comparable with those found in Bolier et al's meta-analysis of controlled PPIs studies in predominantly non-clinical samples [16], but were lower than those in the earlier meta-analysis of Sin and Lyubomirsky [17]. However, in the meta-analysis conducted by Sin and Lyubomirsky [17] less stringent inclusion criteria were used and other interventions such as mindfulness and life-review were included that are commonly not regarded as PPIs [2, 16]. Nonetheless, our findings show promise for PPIs in samples with psychiatric and somatic disorders, and suggest that PPIs, wherein the focus is on eliciting positive feelings, cognitions or behaviors, may also be relevant for clinical populations.

In the field of psychology, especially clinical psychology, the focus lies primarily on examining distress-reducing treatment approaches. As PPIs explicitly aim to improve well-being, the findings of the current study are important because well-being is often impaired in individuals with clinical disorders [23] and low levels of well-being form a substantial risk for relapse or recurrence of symptoms [5, 7]. More importantly, recent studies suggest that well-being and psychological distress are two separate constructs, and that the treatment of symptoms does not necessarily result in improved well-being (e.g., [6, 14]). In the light of these findings, we encourage researchers to further establish the effectiveness of well-being enhancing approaches including PPIs.

Explorative subgroup analyses suggest that guided PPIs are more effective in improving well-being compared to unguided PPIs, such as self-help. Similar findings were found in earlier meta-analytic reviews [16, 17] regarding PPIs in predominantly nonclinical samples, where larger effect sizes were found in therapist-guided interventions (compared with unguided self-help), when the interventions were offered to people with mental health problems. This is also in line with findings regarding supported versus unsupported conventional psychological treatments, such as cognitive behavioral therapy (e.g., [70, 71]) where significant larger effect sizes are observed for supported psychological treatments. Therapist guidance may potentially improve outcomes of PPIs on well-being in samples with psychiatric and/or somatic disorders. However, based on the explorative nature of the subgroup analyses, these findings should be treated with caution and future research should examine the effect of therapist guidance compared to self-help in controlled studies.

No other significant pre-specified moderators of outcome were observed. There was no significant effect of disorder type (i.e. psychiatric vs somatic disorders) and intervention format (i.e. individual vs group). Although, the moderating effect of intervention duration was not significant, the results showed that PPIs with a shorter duration than 8 weeks did not have a significant effect on well-being whereas PPIs with a longer duration had a significant effect on well-being. This finding is in line with earlier meta-analytic reviews [4, 5] and suggests that PPIs are more effective when offered during a longer period of time (more than 8 weeks). In the additional moderator analyses, no significant differences in effect sizes were found for empirically validated PPIs vs other PPIs. For stress, a significantly higher effect size was found for PPIs that had no intervention/waitlist as control condition than for PPIs that had an active control condition or treatment-as-usual as control condition. However, the sample sizes were relatively small in the exploratory subgroup analyses, which limits the interpretation of the differences between groups, and the results should therefore be considered with caution.

The current systematic review and meta-analysis highlights the need to improve the research methodology and reporting within the field of PPIs. The quality of the included studies was low to medium. Although the quality of the studies may have been underestimated since we rated a criterion as not met if it was not reported in the paper, it seems that the methodological quality of studies in this field could be considerably improved if authors routinely report on sequence generation, allocation concealment and blinding of assessors. Furthermore, only one third of the included studies reported using the intention-to-treat principle to analyze the results and almost half of the included studies did not report using a power analysis to determine the sample size. Inadequate statistical power and not adhering to the intention-to-treat principle introduces bias into the results of individual studies, and distorts the results from meta-analyses [72]. This was reflected in the meta-regression analysis which indicated that the effects of PPIs on depression and anxiety were moderated by the methodological quality of the studies, with a lower study quality resulting in smaller effect sizes. Therefore, we recommend researchers conducting studies on PPIs in clinical samples to comply with the quality criteria when designing studies, in order to perform more high quality research to accurately determine the effectiveness of PPIs in clinical samples with psychiatric and somatic disorders. Moreover, the number of studies including post-treatment follow-up measures is relatively low (12 out of 30). We encourage researchers in the field to include follow-up measurements as to determine whether possible favorable effects of PPIs can be sustained in the long run.

Our systematic review and meta-analysis focused on controlled studies of PPIs in clinical samples. We identified a number of studies in different clinical disorders, age groups and settings. Drawing upon these findings in one place has generated the first evidence-based overview of the effectiveness of PPIs in clinical populations. However, several limitations should be noted. One important limitation is that well-being was not always the primary outcome in the included studies. Also, different definitions of well-being were used across the included studies. Incorporating validated measures of well-being, preferably ones that encompass emotional, psychological, and social dimensions of well-being [73], in future studies of PPIs is recommended. Second, the effects of the PPIs may also have been overestimated due to publication bias. Although the results of this meta-analysis point at significant but small effects of PPIs, after adjustment for publication bias, caution is needed. Third, our conclusions are based on the overall effect after the exclusion of outliers, including studies of low quality. When considering only studies of at least medium quality, the effects of PPIs are substantially lower but the sample size of the studies also decreases substantially. Since this is the first study meta-analyzing the effects of PPIs in clinical samples, we based our conclusions on the analyses (i.e. after excluding outliers) with the largest sample size to present a more comprehensive representation of the field. Fourth, we observed a broad range of PPIs in our meta-analysis that varied in delivery mode and intensity. Future research should examine which clinical populations may benefit from PPIs, in terms of type, delivery mode and intensity, and whether there are differential mediators of outcome. Still, this is one of the first meta-analyses in this field providing an overview of PPIs in clinical samples.

## Conclusions

In conclusion, this systematic review and meta-analysis provides evidence that PPIs are effective in improving well-being as well as in alleviating common psychological symptoms, including depression and anxiety, in clinical samples with psychiatric and somatic disorders. At present, the most promising PPIs seem to be those that are guided. Given the growing interest for PPIs in clinical settings [15, 16], it is timely and important to further establish the potential of PPIs in the context of clinical populations using large-scale and methodologically sound trials.

## Additional files

Additional file 1: Search strategy. Full search strategies for Scopus, Pubmed, and PsycINFO (DOCX 13 kb)

Additional file 2: **Figure S1.** Flowchart of the study selection process. PRISMA Flowchart of the study selection process (DOCX 26 kb)

## Abbreviations

AHI: Authentic happiness index
ANX: Anxiety
BAI: Beck anxiety inventory
BDI-II: Beck depression inventory-II
CES-D: Center for epidemiologic studies depression scale
CI: Confidence interval
CID-A: Clinical interview for depression - anxiety subscale
CID-D: Clinical interview for depression - depression subscale
DBT: Dialectical behaviour therapy
DEP: Depression
DTS: Distress tolerance scale
EWBS: Emotional well-being scale
FACIT-SP: Functional assessment of chronic illness therapy - spiritual well-being scale
HADS: Hospital anxiety and depression scale
HADS-A: Hospital anxiety and depression scale – anxiety scale
HADS-D: Hospital anxiety and depression scale - depression scale
HS: The hope scale
LOT-R: Life orientation test – revised
MLQ: The meaning in life questionnaire
*N*_comp_: Number of comparisons
PA: The positive and negative affect scale - positive affect scale
PDS: Posttraumatic stress diagnostic scale
PPI: Positive psychological intervention
PPTI: Positive psychotherapy inventory
PRISMA: Preferred reporting items for systematic reviews and meta-analyses
PSS: Perceived stress scale
PWB: The Ryff scales of psychological well-being
PWBS: Psychological Well-Being Scale
PWI: Personal well-being index
QIDS-SR: Quick inventory of depressive symptomatology, self-report
S: Stress
SCL-90-A: Symptom checklist 90 – anxiety subscale
SCL-90-D: Symptom checklist 90 - depression subscale
SQ-A: Symptom questionnaire - anxiety subscale
STAI: Spielberger state – trait anxiety inventory
SUDS: Subjective units of distress scale
SWB: Subjective well-being
SWBS: Spiritual well-being scale
SWLS: Satisfaction with Life Scale
TAU: Treatment-as-usual
U: Unknown
WB: Well-being
WEMMWB: Warwick-Edinburgh mental well-being scale
