# Supplementary material for: The effect of positive psychology interventions on well-being and distress in clinical samples with psychiatric or somatic disorders: a systematic review and meta-analysis
Source: BMC Psychiatry. 2018 Jun 27;18:211. doi: 10.1186/s12888-018-1739-2 (PMC6020379; doi:10.1186/s12888-018-1739-2)
Supplement: Supplementary file 1 — Search strategy. Full search strategies for Scopus, Pubmed, and PsycINFO (DOCX 13 kb) [file 12888_2018_1739_MOESM1_ESM.docx]

Additional file 1 - Search strategy

Search strategy: Scopus

#1 TITLE-ABS-KEY(({well-being} OR {wellbeing} OR {well being} OR happiness OR happy OR {life satisfaction} OR {satisfaction with life} OR {positive psych*} OR {positive emotion*} OR {positive feeling*} OR {positive cognition} OR {positive behavio*} OR compassion OR optimism OR gratitude OR kindness)

#2 TITLE-ABS-KEY(intervention* OR therap* OR treatment* OR training* OR program* OR exercise)

#3 TITLE-ABS-KEY(symptom* OR disorder* OR illness* OR disease* OR impairment OR clinic*)

#4 TITLE-ABS-KEY(effect* OR effic* OR outcome* OR evaluat*)

#5 TITLE-ABS-KEY(random* OR RCT* OR control* OR non-random* OR pilot* OR condition)

#6 #1 AND #2 AND #3 AND #4 AND #5 (filters: English, article, limit to subject area psychology and social sciences)

Search strategy: PubMed

#1 ("well-being"[tiab] OR happiness OR happy OR “life satisfaction”[tiab] OR “satisfaction with life”[tiab] OR "positive psychology"[tiab] OR "positive emotion"[tiab] OR "positive feeling"[tiab] OR "positive cognition"[tiab] OR "positive behavior"[tiab] OR "positive behaviour"[tiab] OR compassion[tiab] OR optimism[tiab] OR gratitude[tiab] OR kindness [tiab])

#2 (Happiness[Mh] OR Positive Psychology[Mh] OR Well Being[Mh] OR Optimism[Mh] OR Life Satisfaction[Mh] OR Compassion[Mh] OR Optimism[Mh])

#3 (intervention*[tiab] OR therap*[tiab] OR treatment*[tiab] OR training*[tiab] OR program*[tiab] OR exercise[tiab])

#4 (Therapy[Mh] OR Psychotherapy[Mh] OR Training[Mh] OR Exercise[Mh])

#5 (symptom*[tiab] OR disorder*[tiab] OR illness*[tiab] OR disease*[tiab] OR impairment[tiab] OR “clinical sample”[tiab])

#6 (disorder[Mh] OR disease[Mh] OR clinical psychology[Mh])

#7 (effect*[tiab] OR effic*[tiab] OR outcome*[tiab] OR evaluat*[tiab])

#8 (random*[tiab] OR RCT*[tiab] OR control*[tiab] OR non-random*[tiab] OR pilot*[tiab] OR condition[tiab])

#9 #1 OR #2

#10 #3 OR #4

#11 #5 OR #6

#12 #9 AND #10 AND #11 AND #7 AND #8 (filters: English, Adults)

Search strategy: PsycINFO

#1 ("well-being" OR happiness OR happy OR “life satisfaction” OR “satisfaction with life” OR "positive psych*" OR "positive emotion*" OR "positive feeling*" OR "positive cognition" OR "positive behavio*" OR compassion OR optimism OR gratitude OR kindness)

#2 (DE “optimism” OR DE “well being” OR DE “life satisfaction” OR DE “happiness” OR DE “positive psychology” OR DE “gratitude”)

#3 (intervention* OR therap* OR treatment* OR training* OR program* OR exercise)

#4 (DE “Intervention” OR DE “Therapy” OR DE “Psychotherapy”)

#5 (symptom* OR disorder* OR illness* OR disease* OR impairment OR “clinical sample”)

#6 (DE “Symptoms” OR DE “Disorders” OR DE “Clinical Psychology”)

#7 (effect* OR effic* OR outcome* OR evaluat*)

#8 (random*OR RCT* OR control* OR non-random* OR pilot* OR condition)

#9 #1 OR #2

#10 #3 OR #4

#11 #5 OR #6

#12 #9 AND #10 AND #11 AND #7 AND #8 (filter: academic journals, adults, English)’
