# Supplementary material for: The effect of positive psychology interventions on well-being and distress in clinical samples with psychiatric or somatic disorders: a systematic review and meta-analysis
Source: BMC Psychiatry. 2018 Jun 27;18:211. doi: 10.1186/s12888-018-1739-2 (PMC6020379; doi:10.1186/s12888-018-1739-2)
Supplement: Supplementary file 2 — Figure S1. Flowchart of the study selection process. PRISMA Flowchart of the study selection process (DOCX 26 kb) [file 12888_2018_1739_MOESM2_ESM.docx]

## Screening

## Included

## Eligibility

## Identification

Additional records identified through other sources (*n* = 6)

Records after duplicates removed

(*n* = 9308)

Records excluded (*n* = 9069)

Full-text articles excluded (*n* = 71)

No PPI intervention (*n* = 36)

Not clinical sample (*n* = 22)

No control group (*n* = 3)

No relevant outcome measure (*n* = 8)

Insufficient data (*n* = 2)

Articles included in meta-analysis

(*n* = 30)

Records identified through database searching

Pubmed *n* = 5,945

PsycInfo *n* = 2,410

Scopus *n* = 2,531

Full-text articles assessed for eligibility (*n* = 101)

Titles screened (*n* = 9308)

Abstracts excluded (*n* = 138)

No PPI intervention (*n* = 71)

Not clinical sample (*n* = 33)

No control group (*n* = 28)

Not adult sample (*n* = 3)

No relevant outcome measure (*n* = 3)

Abstracts screened (*n* = 239)
